# Hyperhomocysteinemia Is a Result, Rather than a Cause, of Depression under Chronic Stress

**DOI:** 10.1371/journal.pone.0106625

**Published:** 2014-10-06

**Authors:** Shen Chengfeng, Liu Wei, Wang Xinxing, Wu Lei, Zhan Rui, Qian Lingjia

**Affiliations:** 1 Tianjin Centers for Disease Control and Prevention, Tianjin, China; 2 Beijing Institute of Basic Medical Sciences, Beijing, China; 3 Institute of Health & Environmental Medicine, Tianjin, China; Xi'an Jiaotong University School of Medicine, China

## Abstract

**Background:**

Although the accumulation of homocysteine (Hcy) has been implicated in the pathogenesis of depression, whether Hcy is directly involved and acts as the primary cause of depressive symptoms remains unclear. The present study was designed to clarify whether increased Hcy plays an important role in stress-induced depression.

**Results:**

We employed the chronic unpredictable mild stress model (CUMS) of depression for 8 weeks to observe changes in the plasma Hcy level in the development of depression. The results showed that Wistar rats exposed to a series of mild, unpredictable stressors for 4 weeks displayed depression-like symptoms such as anhedonia (decreased sucrose preferences) and a decreased 5-Hydroxy Tryptophan (5-HT) concentration in the hippocampus. At the end of 8 weeks, the plasma Hcy level increased in the CUMS rats. The anti-depressant sertraline could decrease the plasma Hcy level and improve the depression-like symptoms in the CUMS rats. RhBHMT, an Hcy metabolic enzyme, could decrease the plasma Hcy level significantly, although it could not improve the depressive symptoms in the CUMS rats.

**Conclusions:**

The results obtained from the experiments did not support the hypothesis that the increased Hcy concentration mediated the provocation of depression in CUMS rats, and the findings suggested that the increased Hcy concentration in the plasma might be the result of stress-induced depression.

## Background

Depressive disorders are among the most frequent types of mental illnesses. Stress is involved in the etiology of depression [1]. Although dysfunction of the hypothalamic-pituitary-adrenal (HPA) axis and sympathetic nervous system, associating stress with depression, have been discussed, the pathological mechanisms remain unclear. Homocysteine (Hcy) is a thiol amino acid that is generated from the metabolism of methioline, which has been associated with many diseases such as cardiovascular disease and depression [2]–[4]. High concentrations of Hcy in the plasma or serum have been found in depressive patients. It was reported that approximately 20–50% of patients with severe depression had increased total Hcy levels in the plasma [5], [6]. Furthermore, Tolmunen et al. [7] determined in a cross-sectional study that the subjects in the upper tertile for serum total Hcy had a more than two-fold higher risk of being depressed compared with the subjects in the lowest tertile for serum total Hcy. A further study confirmed that being in the lowest quartile of Hcy was associated with fewer depressive symptoms after adjusting for sex, physical health, smoking, and other variables. A high level of Hcy correlates to depressive symptoms in community-dwelling middle-aged individuals [8]. Therefore, as demonstrated by the epidemiological studies described above, an elevated Hcy concentration in plasma is very common in depression. Although the accumulation of Hcy has been implicated in the pathogenesis of depression [9], whether Hcy is directly involved and acts as primary cause of depressive symptoms is unclear [10], and there is little evidence from animal experiments.

The Hcy level in plasma or serum could be influenced by many factors such as age, vitamin deficiency, renal function, and a common mutation in the methylenetetrahydrofolate reductase (MTHFR) gene that impairs Hcy metabolism. Subjects with the TT genotype have a higher Hcy level [11]. Aside from the factors mentioned above, Stoney et al.. demonstrated that acute psychological stress could induce rapid and significant elevations in the plasma total Hcy concentration in a sample of women between 40 and 63 years of age [12]. Oliveira et al.. found that with exposure to restraint stress, a model of psychological stress in rats, the total plasma Hcy concentration increased significantly [13]. Our previous results supported this finding. Setnika reported that foot shock stress also increased the plasma Hcy in female rats that were in either estrus or diestrus, although it did not increase the plasma Hcy in male rats [14]. These studies indicated that the Hcy concentration might be affected by some stressful manipulations.

Because stress has been thought to be one of etiological factors in depression [1], we suspected that hyperhomocysteinemia is a result or a cause of depression induced by stress. To demonstrate this hypothesis, we first established a rat stress model of depression using the chronic unpredictable mild stress (CUMS) described by Willner and colleagues [15]; we then observed the depression-like symptoms and the changes in the plasma Hcy level in CUMS rats and evaluated the effects of the antidepressant sertraline on the depression-like symptoms and the changes in the Hcy level in CUMS rats. These results would be significant for the understanding of the mechanism of stress-induced depression and could provide information regarding the treatment of depression.

## Methods

### Agents

The following materials were used: 7-fluorobenzene-2-oxy-1, 3-diazolic-4-ammonium sulfate–SBD-F (SBD-F, sigma, USA), N-acetyl-L-cysteine (NAC, sigma, USA), serotonin (5-HT, sigma, USA), dihydroxybenzylamine (DHBA, sigma, USA), and sertraline hydrochloride tablets purchased from HuiRui Inc, China. All of the other reagents used were of analytical or chromatographic grade.

### Animals

This study was conducted with the approval of the Animal Care and Use Committee of the Beijing Institute of Basic Medical Sciences, China. The protocol was approved by the Committee on the Ethics of Animal Experiments of the Beijing Institute of Basic Medical Sciences (Permit Number: 2012-D-3098). All of the surgical procedures were performed under sodium pentobarbital (1%, 1 ml/100 g weight, i.p.) anesthesia, and efforts were made to minimize suffering.

Male Wistar rats (200–220 g, Tianjin, China) were used for the experiment, and 8 rats were treated in each group. The rats were acclimated to the surroundings for 1 week prior to the start of experiment. The animals were maintained on a 12 h light/dark cycle under a controlled temperature of 25±2°C. Food and water were available for the duration of the experiments unless otherwise noted. All of the animal handling procedures were performed in strict accordance with the guide for the use and care of laboratory animals.

### Sucrose Preference Tests and Body Weight Measurements

Anhedonia, which is a central feature of depression, was defined as a reduction in sucrose preference. The methods were similar to those described by Muscat and Willner et al. [15]–[17]. Rats were trained for adaptation to the taste of a sucrose solution (1%) prior to the start of the experiment. After removing the food and water from each rat's cage for a period of 20 hours, the rats were exposed to water and a 1% sucrose solution in pre-weighed plastic bottles, which were placed on the cages; the animals were allowed to consume the fluids for a period of 1 h. The bottles were then removed and weighed to determine the amount (in grams) of fluid consumed by rats. The tests were conducted, and the body weights were measured before commencing the CUMS protocol (baseline) and every 2 weeks for 8 weeks. Sucrose preference was calculated according to the following formula: %sucrose preference = (sucrose intake/total fluid intake)×100.

### Protocol of Chronic Unpredictable Mild Stress (CUMS)

CUMS is employed to mimic negative life events to which human beings are exposed and to produce anhedonia in rats, which is an important indicator of the establishment of the CUMS animal model [15]–[18]. According to the rat sucrose preference in the baseline test, the rats were divided into 2 groups: control and CUMS animals. Except for the control rats, the other animals were kept in individual cages. The experimental protocol and CUMS procedure were a variation of methods described by Grippo et al. [19], [20]. The CUMS procedure was carried out for a total of 8 weeks. The stressors for CUMS include continuous overnight illumination, 40°C cage tilt along the vertical axis, paired housing, soiled cage (200 ml water spilled into the bedding of each cage), restraint in a small cage (equipped with breathing holes), exposure to an empty water bottle for 1 hour immediately following a period of acute 1-hour water deprivation, and food deprivation. All of the stressors were applied individually and continuously, day and night. The control animals were left undisturbed in their home cages with the exception of general handling (i.e., regular cage cleaning and measuring body weight).

### Open field test

Locomotor activity and exploratory behavior were evaluated using an open field test. The open field apparatus was a plywood box measuring 100 cm ×100 cm. All of the walls were painted black. The floor was divided into 25 equal squares. The rats were placed individually in one corner of the apparatus, and their ambulation (number of squares crossed) and immobility frequency were observed for 3 m. An open field test score was calculated as the sum of ambulation and immobility frequency [21].

### Forced Swimming Test

A forced swimming test was performed in accordance with the method of Elizete et al.. [22]. The test was performed after 24 hours of treatment. The rats were placed into a cylinder with a diameter of 40 cm containing a column of 17 cm of water at 27°C. The animals were trained 24 hours before the forced swimming test. Then, the rats were exposed to the same experimental conditions for 5 min. A rat was judged to be not moving if it remained floating in the water, in an upright position, with only a small amount of movement to keep its head above water. The immobility time was recorded.

### Pharmacological treatment

According to their sucrose preference following 4 weeks of CUMS, the rats were divided into eight groups; a control group that was orally administered water, a control group that received sertraline hydrochloride (Po, 10 mg/kg·day), a control group that received RhBHMT (IV, 0.04 mg/kg·day), a control group that received L-methionine (IG, 1 g/kg·day), the CUMS group, a CUMS with sertraline-treated group, a CUMS with RhBHMT-treated group and a CUMS with L-methionine-treated group. Sertraline is a selective serotonin reuptake inhibitor (SSRI) and appears to be a safe and efficacious treatment for depression [23]–[25]. RhBHMT is a recombinant human betaine-homocysteine S-methyltransferase that could promote Hcy methyltransferase metabolism specifically and decrease the plasma Hcy level. The CUMS procedures were performed for a total of 8 weeks.

### Preparation of Blood Samples and Tissue Collection

Before the initiation of the experiment and every two weeks for 8 weeks, blood was collected from the medial angle of the rat eye into precooled plastic vials containing 0.2 ml of an EDTANa_2_ solution (60 mg/ml) and centrifuged at 900 g for 20 min at 4°C. The plasma was extracted, transferred to plastic tubes and stored at −80°C until the Hcy assays were performed [13], [26].

Immediately after the body weight measurements and sucrose preference tests, all of the animals were left without any treatment until the following morning. They were brought into an adjacent room and then decapitated, and the whole brains were quickly removed onto an ice-plate and washed with 0.9% saline solution. The hippocampus was carefully dissected for the 5-HT assays.

### Determination of 5-HT in the hippocampus

The 5-HT levels were determined in the hippocampus using a modification of previous methods [27]. Briefly, the brain tissues were homogenized with an ultrasonic homogenizer in an ice-cold solution (15 µl/mg tissue) of 0.09 M perchloric acid containing 5 mM sodium bisulfite, 0.04 mM EDTA and dihydroxybenzylamine as the internal standard. The homogenate was then centrifuged at 14000× *g* for 20 min at 4°C. The obtained supernatant was subjected to chromatography on a C18 model column (4.6 mmi.d.×250 mm, 10 µm microparticles). The chromatography system consisted of an apparatus and a LC6A electrochemical detector. The measurements were performed at an electrode potential of +0.7 V versus the Ag/AgCL reference electrode.

### Measurement of the plasma Hcy concentration

The total plasma Hcy values were determined using high-performance liquid chromatography (HPLC) with fluorimetric detection and isocratic elution [28]. This method involves the reduction of thiol groups, protein precipitation and derivatization with 7-fluorobenzene-2-oxy-1, 3-diazolic-4-ammonium sulfate–SBD-F. The HPLC system included a WATERS LC2695 apparatus and a WATERS 2475 fluorescence detector. Chromatographic separation was performed using a C18 model SymmetryShield RP18 column (3.9 mmi.d.×150 mm, 5 µm microparticles). The fluorescence of the separated compounds was detected with a detector adjusted for excitation at 390 nm and emission at 470 nm. The Hcy content was calculated with a calibration curve using a known Hcy concentration and N-acetyl-L-cysteine (NAC) as the internal standard.

### Statistical analyses

The values are presented as the means ± SE for the indicated experiments and were analyzed by mixed-design ANOVA, one-way ANOVA and Student's *t*-tests, where appropriate. A probability value of *P*<0.05 was considered statistically significant.

## Results

### Unpredictable chronic mild stress induces the comorbidity of depression-like behavior and hyperhomocysteinemia in rats

**Figure 1:**
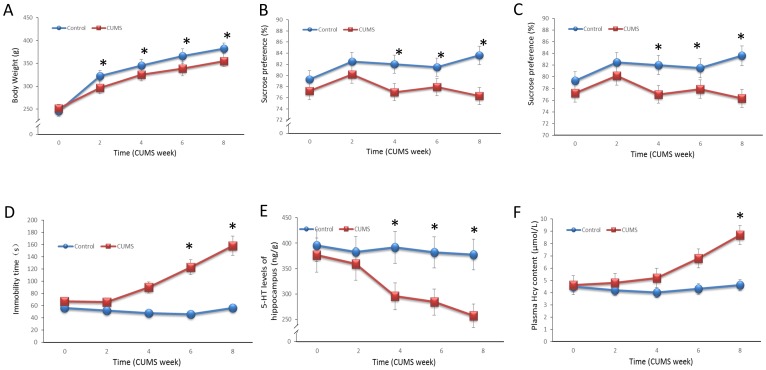
Unpredictable chronic mild stress induces the comorbidities of depression-like behavior and hyperhomocysteinemia in rats. The depression-like behavior of CUMS rats was measured at 2, 4, 6 and 8 weeks. **A.** The rats were weighed weekly, and the weights are shown. **B.** A 2-bottle sucrose preference test was used to measure the rats' preferences for sweetened solution over water. Preference was calculated as the percentage of sucrose solution consumed compared with the total fluid intake: %sucrose preference = (sucrose intake/total fluid intake)×100. **C.** Exploratory behavior was evaluated as described in the Materials and Methods section. **D.** The immobility time was detected by manual measurements. **E.** 5-HT level in the hippocampus was detected using liquid chromatography (Waters E2695). **F.** The total plasma Hcy values were determined by high-performance liquid chromatography (HPLC) with fluorimetric detection and isocratic elution. Values represent the group mean ± structural equation modeling (SEM) (n = 8 rats per group). *P<0.05 compared with control, repeated measures ANOVA followed by Tukey's multiple comparison tests.

The changes in the body weight in the control and CUMS groups across time (baseline through 8 weeks CUMS) are shown in Fig. 1A. The analysis yielded a main effect of time [F(4,110) = 271.89, *P*<0.001], group [F(1,110) = 52.78, *P*<0.001] and a significant group by time interaction [F(4,110) = 5.86, *P*<0.001]. After 2 weeks of CUMS throughout the protocol, significantly decreased body weights in the CUMS rats were observed compared with the decreases in the control groups (Week 2: *P* = 0.003, Week 4: *P* = 0.017, Week 6: *P* = 0.002, Week 8: *P*<0.001). The sucrose preference test was used to evaluate anhedonia, which is the most important index for the establishment of the CUMS model. As shown in Fig. 1B, an ANOVA yielded significant main effects of group [F(1,98) = 11.41, *P* = 0.001]. A comparison of the means in sucrose preference revealed a significant difference between the control and CUMS groups at 4, 6 and 8 weeks (*P* = 0.049, *P* = 0.048, *P* = 0.001, respectively), which indicated that the rats in the CUMS group showed depression-like behavior. The total open field test scores are shown in Fig. 1C. The open field test score of the CUMS rats decreased significantly compared with the score of the control group after 4, 6 and 8 weeks of CUMS [F(2,73) = 7.42, *P* = 0.001]. As shown in Fig. 1D, the immobility time of the CUMS rats was increased significantly after 6 and 8 weeks of CUMS [F(2,71) = 8.36, *P*<0.001].

Changes of the 5-HT level in different brain areas were typically found in the CUMS rats, which supported the establishment of a CUMS depression model. Fig. 1E presents the 5-HT concentrations of the hippocampus of both groups. An ANOVA was performed on the 5-HT level in the hippocampus. Consistent with our expectations, the analysis yielded significant main effects of group [F(1,20) = 26.66, *P*<0.001], which indicated a decreased 5-HT concentration in the hippocampus in the CUMS rats after 4 weeks of CUMS (Week 4: *P* = 0.003, Week 8: *P* = 0.007).

The plasma Hcy concentrations are displayed in Fig. 1F. An ANOVA yielded significant main effects of group [F(1,40) = 13.66, *P*<0.001]. After exposure to CUMS for 6 weeks, the plasma Hcy concentration in the CMS group increased in the CUMS rats; however, this result did not reach statistical significance (*P*>0.05). At the end of the 8-week CUMS exposure, the plasma Hcy concentration was significantly increased [*t*(10) = 13.66, *P* = 0.004].

### Sertraline inhibits depression-like behavior and the plasma Hcy concentration in CUMS rats

**Figure 2:**
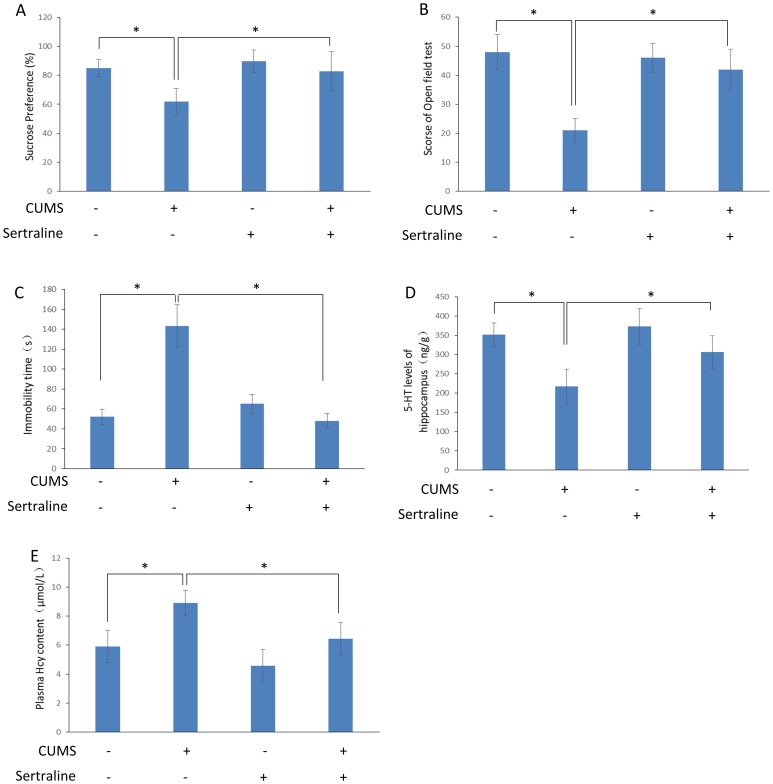
Sertraline inhibits depression-like behavior and plasma Hcy content in CUMS rats. Sucrose preference, open field test scores, 5-HT level in the hippocampus and total plasma Hcy were measured as described in Figure 1. **A.** Sertraline inhibited the decline of sucrose preference induced by CUMS. **B.** Sertraline inhibited the decline of the open field test score induced by CUMS. **C.** Sertraline decreased the immobility time of the forced swimming test induced by CUMS. **D.** Sertraline inhibited the decline in the 5-HT level in hippocampus induced by CUMS. **E.** Sertraline inhibited the increase in the plasma Hcy level induced by CUMS. Values represent the group mean ± structural equation modeling (SEM) (n = 8 rats per group). *P<0.05, repeated measures ANOVA followed by Tukey's multiple comparison tests.

The alterations in the sucrose preference, total open field test scores, 5-HT level in the hippocampus and plasma Hcy concentration after CUMS following sertraline treatment are analyzed in Fig. 2. At the end of 8 weeks of CUMS, significant differences in the sucrose preference, total open field test scores and 5-HT level of the hippocampus between the groups were revealed [sucrose preference: F(3,36) = 17.813, *P*<0.001, total open field test scores: F(3,26) = 16.582, *P*<0.001, 5-HT level: F(3,20) = 16.357, *P*<0.001]. The sucrose preference, total open field test scores, immobility time of the forced swimming test and 5-HT level of the hippocampus in the CUMS+sertraline rats were increased compared with the CUMS rats [sucrose preference: *t*(19) = 4.26, *P*<0.001, total open field test scores: *t*(13) = 4.35, *P*<0.001, Immobility time: *t*(12) = 3.86, *P*<0.001, 5-HT level: *t*(10) = 3.53, *P* = 0.005]. The plasma Hcy concentration between the groups was significantly different [F(3,20) = 17.115, *P*<0.001]; this concentration was lower in the CUMS+sertraline rats compared with the CUMS rats [*t*(10) = 4.23, *P* = 0.002].

### RhBHMT suppresses the plasma Hcy level, although it has no effect on depression-like behavior

**Figure 3:**
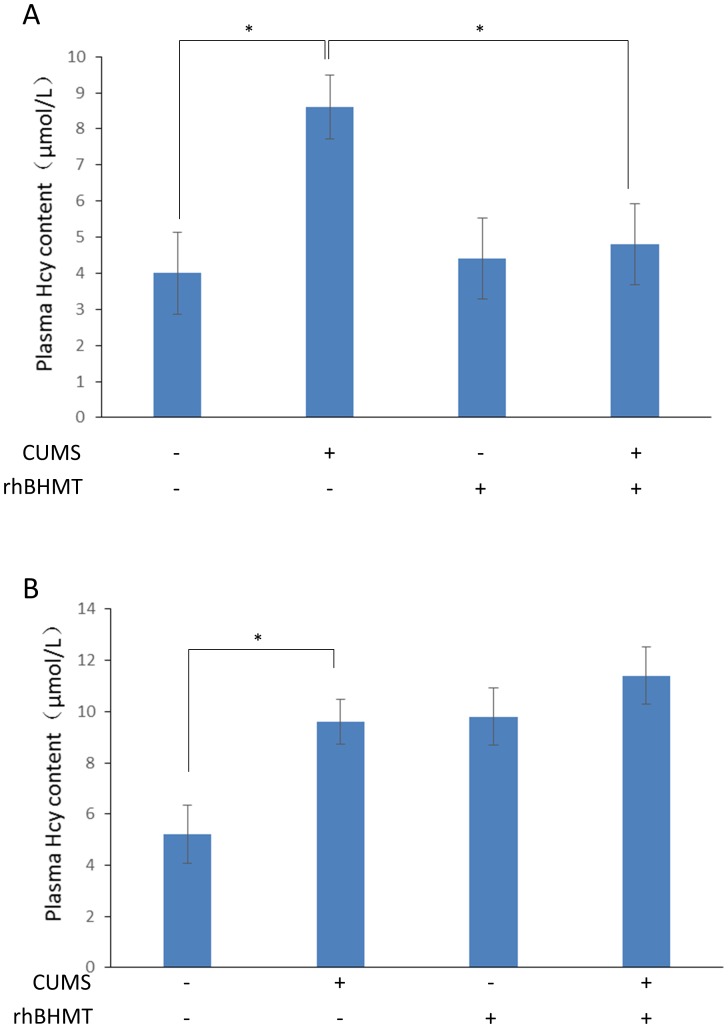
RhBHMT suppress plasma Hcy level in CUMS rats. Total plasma Hcy levels were measured as described in the Methods section. **A.** RhBHMT inhibited the plasma Hcy level induced by CUMS rats. **B.** Methionine increased the plasma Hcy level compared with the control group. Values represent the group mean ± structural equation modeling (SEM) (n = 8 rats per group). *P<0.05, repeated measures ANOVA followed by Tukey's multiple comparison test.

**Figure 4:**
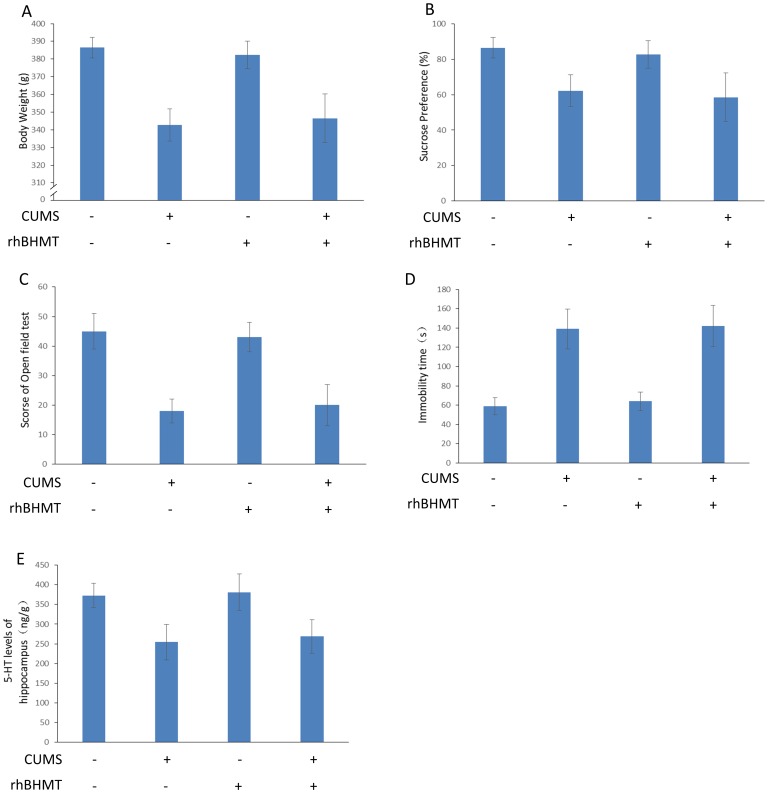
Effect of RhBHMT on depression-like behavior in CUMS rats. Body weight, sucrose preference, open field test scores, immobility time of the forced swimming test, 5-HT level in the hippocampus and total plasma Hcy were measured as described in Figure 1. RhBHMT had no effect on the body weight, sucrose preference, open field test scores, immobility time of the forced swimming test and 5-HT level in the hippocampus of the CUMS rats. Values represent the group mean ± structural equation modeling (SEM) (n = 8 rats per group). *P<0.05, repeated measures ANOVA followed by Tukey's multiple comparison test.

The changes in the Hcy level in the plasma are described in Fig. 3. At the end of 8 weeks of CUMS, a significant difference in the plasma Hcy concentration was revealed [F(3,27) = 16.723, *P*<0.001]. RhBHMT is a recombinant human betaine-homocysteine S-methyltransferase. In our previous study, we demonstrated that RhBHMT could effectively suppress the level of plasma Hcy in hyperhomocysteinemia rats induced by a methionine diet. In the present study, the plasma Hcy concentration in the CUMS+RhBHMT group rats were decreased compared with the CUMS rats [t(14) = 3.98, *P*<0.001]. The effects of RhBHMT on the body weight, open field test, sucrose preference and 5-HT level of the hippocampus are shown in Fig. 4. However, RhBHMT could not improve the depressive symptoms in the CUMS rats. These results illustrate that reducing the plasma Hcy level had no significant effect on depression-like behavior of rats exposed to CUMS. The Hcy level of plasma was determined during the 24-h period (measured every 4 hours) after RhBHMT treatment (Fig. S1). The Hcy level was not significantly increased in the RhBHMT intervention gap.

### Methionine treatment does not have an effect on depression-like behavior

**Figure 5:**
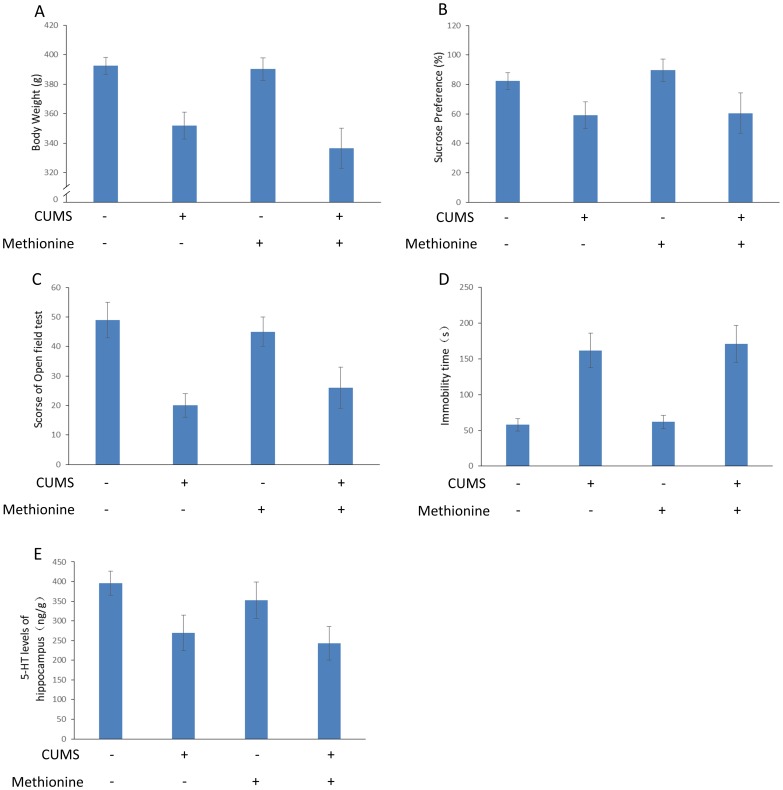
Effect of methionine on depression-like behavior in CUMS rats. Body weight, sucrose preference, open field test scores, immobility time of the forced swimming test, 5-HT level in the hippocampus and total plasma Hcy were measured as described in Figure 1. Methionine had no effect on the body weight, sucrose preference, open field test scores, immobility time of the forced swimming test and 5-HT level in the hippocampus of CUMS rats. Values represent the group mean ± structural equation modeling (SEM) (n = 8 rats per group). *P<0.05, repeated measures ANOVA followed by Tukey's multiple comparison tests.

The changes in the Hcy level in the plasma are described in Fig. 3. Methionine treatment increased the level of Hcy in the plasma significantly compared with the control group [t(13) = 4.39, *P*<0.001]. The effects of methionine on the body weight, open field test, sucrose preference, immobility time of the forced swimming test and 5-HT level of the hippocampus are shown in Fig. 5. Methionine did not have an effect on the depressive symptoms in CUMS rats.

## Discussion

As supported by some studies, depression is association with an increased Hcy concentration, which could be affected by some stressful manipulations. The present study attempted to elucidate whether the increased Hcy concentration mediated the association underlying stress and depression. The results demonstrated that an increased plasma Hcy level was found at the end of 8 weeks of CUMS exposure; the results did not support the hypothesis that the increased Hcy concentration was one of possible intermediate mechanisms for the provocation of CUMS-induced depression because decreased sucrose preference following CUMS 4 weeks occurred prior to the increase of plasma Hcy concentration. Our study also demonstrated that the anti-depressant sertraline could decrease the plasma Hcy level while improving the depression-like symptoms in this stress model of depression, which indicated that the increased Hcy concentration might be the result of depression in the CUMS model.

The CUMS model is available to the study the pathogenesis, biological mechanisms, and treatments for depression and is focused on the behavioral sign of anhedonia (a central feature of depression characterized by an impaired responsiveness to pleasurable stimuli), which could be examined by sucrose preference tests [17], [18], [29]. In the present study, the rats displayed reduced sucrose preference after exposure to CUMS for 4 weeks, and this result reflected the occurrence of depression-like behavior, which is similar to previous studies [16], [19], [20]. Although decreased body weight was observed, it was previously reported that there was no significant relationship between the effects of CUMS on sucrose intake and decreases in body weight [30]. The 5-HT level in the hippocampi were reduced in the CUMS rats, which also supported the occurrence of depression [31].

Regarding the Hcy assay, we expected to see elevated plasma Hcy levels in CUMS rats compared with control groups, and our results were consistent with this expectation. Modest increases in the plasma Hcy levels in the rats exposed to CUMS 6 weeks were found; however, this increase did not reach statistical significance. The plasma Hcy level at the end of 8 weeks of CUMS increased significantly, and this increase occurred after depression-like behavior appeared in the CUMS rats. Some studies reported that there was no association between an increased Hcy level and the propensity of developing depressive behavior [32], [33]. However, according to some investigations, the thermolabile variant of MTHFR was significantly more common in the group with a history of depressive disorders. The higher Hcy level induced by the MTHFR C677T genotype variant is associated with an increased risk of depressive episodes [34]. Kohen et al.. [35] reported that in a prospective clinical trial, folic acid supplementation could significantly improve the cognitive and depressive status test scores of the majority of volunteers. This result might be related to the fact that folic acid supplementation could lower the Hcy levels. However, these effects were not observed in the present study, which was consistent with the results of Watanabe et al.. They determined that there was no relationship among folate, homocysteine levels and depression in women during early pregnancy [36]. The reasons for these inconsistencies are unclear. Tiemeier reported that **t**he higher rates of depressive disorders in subjects with high Hcy levels are due to differences in cardiovascular factors and physical comorbidities [33]. The results in the current study appeared to reflect that the high plasma Hcy level was the result of depression, although it was not the reason, or that depression-like changes and elevated plasma levels were produced by the common pathological mechanisms initiated by CUMS.

To clarify these uncertainties, the anti-depressant sertraline [25] and the anti-hyperhomocysteinemia agent RhBHMT were employed. The results demonstrated that sertraline improved the depression-like behavior and also decreased the elevated plasma Hcy concentration in the CUMS rats. However, RhBHMT only suppressed the plasma Hcy level and had no effect on the depression-like behavior. This result suggested that the increased Hcy concentration in plasma might be the result of, although not the cause of, depression. In recent reports of investigational observations, the effects of SSRI antidepressants on immune function and the effects of anticytokine pharmacological agents were demonstrated [37]. However, the conclusion of a direct effect of sertraline on Hcy metabolism could not be drawn. This study could not rule out the possibility either. Further research about how sertraline affects the plasma Hcy level is needed. An increase in the total Hcy plasma concentration (the sum of protein-bound, free and disulfide fractions) is now recognized as an risk factor for many diseases, such as cardiovascular disease [3], [4]. The present study also demonstrated that a high Hcy concentration might be an intermediate factor between depression and other diseases. Considering the damaging effect of a high Hcy concentration, it is very important to decrease the elevated plasma Hcy concentration in patients with depression in clinical treatment; sertraline and RhBHMT might be the drugs for the treatment of depressive patients with high plasma Hcy levels.

## Conclusions

The anti-depressant sertraline could decrease the plasma Hcy level while improving the depression-like changes in the CUMS rats. However, RhBHMT suppressed the plasma Hcy level, although it had no effect on the depression-like behavior. These findings might help clarify the mechanisms underlying the association between increased Hcy concentration and stress-induced depression; the results did not support the hypothesis that the increased Hcy concentration mediated the provocation of depression in CUMS rats. The results suggested that the increased Hcy concentration in plasma might be the result of depression in CUMS rats.

## Supporting Information

Figure S1 **Effect of rhBHMT on total plasma Hcy in CUMS rats.** Total plasma Hcy were measured as described in Figure 1. The Hcy level of plasma was determined during the 24-h period (measured every 4 hours) after RhBHMT treatment. The Hcy level was decreased after 4 hours and not significantly increased in the RhBHMT intervention gap. Values represent the group mean ± structural equation modeling (SEM) (n = 8 rats per group). *P<0.05 compared with control, repeated measures ANOVA followed by Tukey's multiple comparison tests.
(TIF)
