# Supplementary figures and images for: Hyperhomocysteinemia Is a Result, Rather than a Cause, of Depression under Chronic Stress
Source: PLoS One. 2014 Oct 6;9(10):e106625. doi: 10.1371/journal.pone.0106625 (PMC4186820; doi:10.1371/journal.pone.0106625)

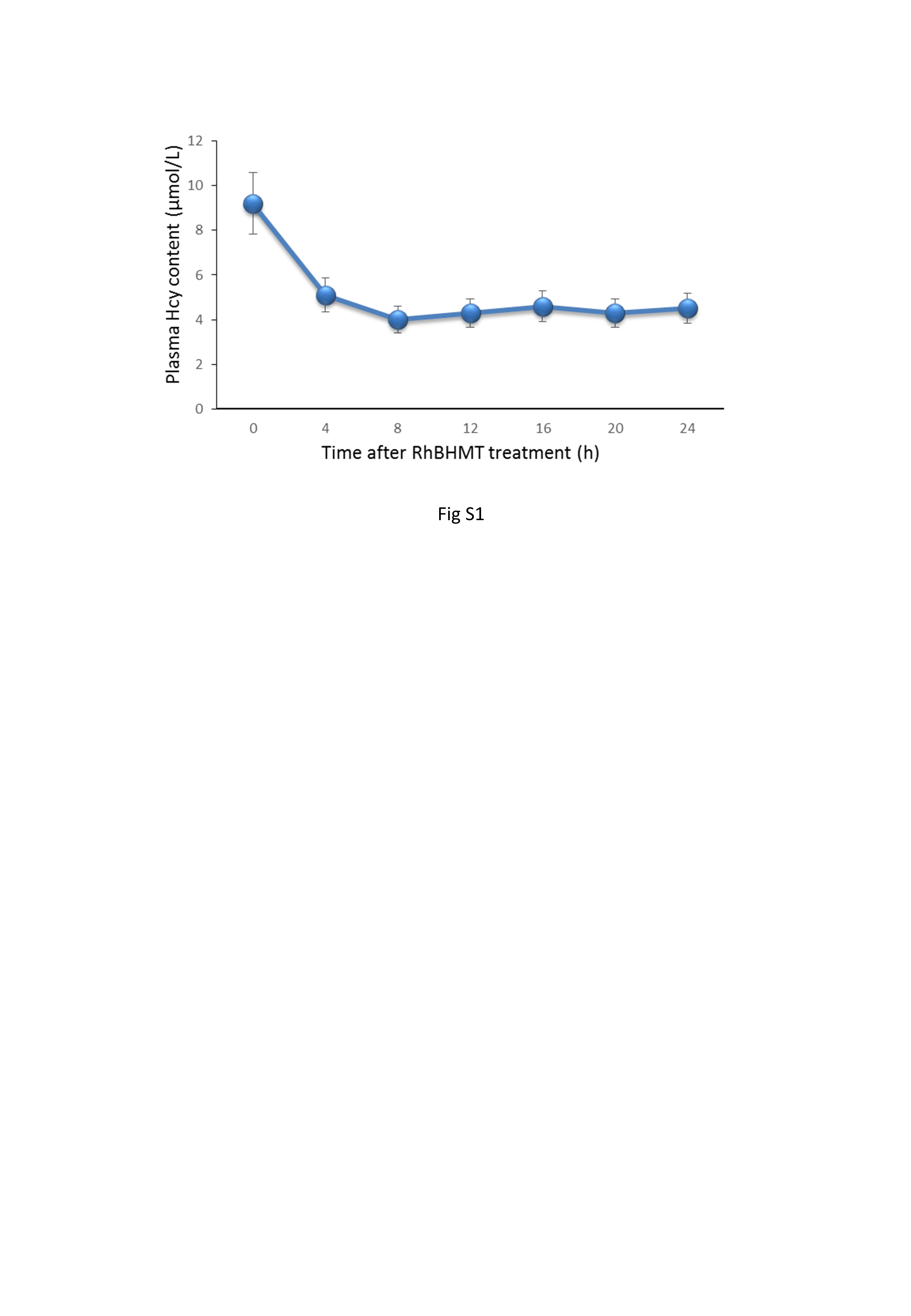

Supplement: Figure S1 — Effect of rhBHMT on total plasma Hcy in CUMS rats. Total plasma Hcy were measured as described in Figure 1. The Hcy level of plasma was determined during the 24-h period (measured every 4 hours) after RhBHMT treatment. The Hcy level was decreased after 4 hours and not significantly increased in the RhBHMT intervention gap. Values represent the group mean ± structural equation modeling (SEM) (n = 8 rats per group). *P<0.05 compared with control, repeated measures ANOVA followed by Tukey's multiple comparison tests. (TIF) [file pone.0106625.s001.tif]
